# Identification of Methylstat as a Potential Therapeutic Agent for Human Glioma Cells by Targeting Cell Cycle Arrest

**DOI:** 10.3390/ph18091344

**Published:** 2025-09-08

**Authors:** Haoge Yao, Tingyi Meng, Yingying Yang, Huaping Tao, Wenwen Lu, Mingqi Liu, Xiaofeng Zhao, Mengsheng Qiu, Aifen Yang

**Affiliations:** 1 Zhejiang Key Laboratory of Organ Development and Regeneration, College of Life and Environmental Sciences, Hangzhou Normal University, Hangzhou 311121, China; 2023111010015@stu.hznu.edu.cn (H.Y.); 2023111010065@stu.hznu.edu.cn (T.M.); 2021111010044@stu.hznu.edu.cn (Y.Y.); msqiu@hznu.edu.cn (M.Q.); 2 Key Laboratory of Specialty Agri-Product Quality and Hazard Controlling Technology of Zhejiang Province, College of Life Sciences, China Jiliang University, Hangzhou 310018, China; 07a0904048@cjlu.edu.cn

**Keywords:** glioma, cell cycle, apoptosis, methylstat, theabrownin

## Abstract

**Background/Objectives**: Glioblastoma (GBM) is the most common and aggressive primary brain tumor in adults, with a poor prognosis and limited therapeutic options. This study aimed to repurpose methylstat, a selective histone demethylase inhibitor, as a novel anti-glioma agent. We characterized its anti-proliferative efficacy, elucidated mechanisms of cell cycle regulation, and evaluated its blood–brain barrier (BBB) permeability potential. **Methods**: Compounds with transcriptional profiles enriched for cell cycle arrest and tumor-suppressive pathways were identified via Connectivity Map (CMAP) analysis. Methylstat was selected based on its high connectivity score and favorable physicochemical properties. In vitro assays were performed to evaluate its effects on cell viability, proliferation, cell cycle progression, and expression of related molecular markers in U251 and HOG glioma cell lines. Molecular docking and 200 ns molecular dynamics (MD) simulations were performed to evaluate the binding mode and stability of the Methylstat–JMJD2A complex. An in vitro BBB model was established to assess the ability of Methylstat to cross the BBB. **Results**: Methylstat significantly inhibited glioma cell proliferation in a dose-dependent manner without inducing apoptosis. It caused G1-phase arrest in U251 cells and G2-phase arrest in HOG cells. Mechanistically, methylstat downregulated cyclins and cyclin-dependent kinases via the p53/p21 pathway. Additionally, methylstat reduced the expression of JMJD2A and its downstream targets, including PDK1, AKT, and mTOR. Molecular docking studies and 200 ns MD simulations confirmed the stable binding of methylstat to the catalytic pocket of JMJD2A, effectively inhibiting its enzymatic activity. HPLC analysis confirmed that methylstat could penetrate the in vitro BBB model to varying extents. **Conclusions**: Methylstat is a promising small-molecule agent that effectively suppresses glioma cell growth by modulating key cell cycle regulators. Its ability to cross the BBB highlights its potential as a novel therapeutic strategy for GBM and other brain tumors.

## 1. Introduction

Gliomas stand as the most prevalent primary brain tumors, contributing significantly to high mortality and recurrence rates. Among them, glioblastoma (GBM) is the most aggressive form, accounting for approximately 48% of all primary malignant tumors in the central nervous system (CNS) [1]. GBM is highly invasive, infiltrating normal brain parenchyma and posing substantial challenges for effective treatment. Currently, the standard treatment for glioma involves initial surgical resection, followed by chemotherapy, or radiotherapy, depending on tumor type, molecular characteristics, and clinical guidelines [2]. However, management of glioma is plagued by limitations such as low cure rates, high postoperative recurrence rates, unsatisfactory prognoses, and multidrug resistance [3]. In recent years, novel therapeutic strategies for GBM have emerged, enabling more precise, mechanism-based approaches by targeting specific cells and tumor-associated antigens [4,5]. These include advanced radiotherapies [6,7], tumor-treating fields [8], molecularly targeted agents [9,10,11,12], immunotherapies [13,14,15,16], and emerging CRISPR/Cas9 gene-editing technologies [17]. Nevertheless, clinical translation remains constrained by challenges such as limited blood–brain barrier (BBB) penetration, on-target off-tumor mitigation toxicity, high costs, variable antigen expression, tumor immune evasion, and significant neurological or systemic side effects [9,18,19]. As a result, the discovery of novel agents with improved brain delivery, mechanistic diversity, and robust pharmacological profiles is crucial for advancing glioma treatment.

Our previous research has revealed that theabrownin (TB) inhibits cell proliferation through the caspase3-dependent and PTEN/AKT pathways, promotes apoptosis in glioma, and specifically induces cell cycle arrest in glioma cells of different origins via c-Myc and p53 [20]. Several prospective studies have shown a significant negative correlation between tea consumption and the risk of glioma [21,22]. Recent studies have provided deeper insights into the chemical composition and structural complexity of TB. Advanced analytical techniques have identified a range of bioactive constituents within TB, including jaceosidin, triethyl citrate, α-naphthoflavone, epicatechin, voriconazole, quercetin, apigenin 7-rutinoside, and dodecylbenzenesulfonic acid [23]. These findings highlight the heterogeneous and polymeric nature of TB, suggesting that its biological effects arise from the synergistic interplay of multiple constituents, potentially fine-tuning cellular pathways pertinent to glioma treatment. However, as a complex macromolecular compound, TB presents significant challenges in isolating and purifying the active substance. Moreover, its precise structure characteristics and key physicochemical properties remain elusive, which hinders in vivo studies on the molecular mechanisms of theabrownin in glioma. Therefore, screening and investigating alternative monomeric drugs to TB for glioma treatment is a topic worthy of further exploration.

The Connectivity Map (CMAP, version 1.1.1.43) is a comprehensive database that establishes correlations between small molecule drugs, gene expression and diseases. It is based on millions of gene expression signatures obtained from millions of perturbation treatments [24]. Users can leverage the differentially expressed signals in cells caused by gene perturbation to identify drugs highly associated with disease and screen new compounds for specific targets through pattern matching algorithms and databases [25]. The CMAP has proven instrumental in predicting promising therapeutic candidates for a wide range of diseases, including osteoarthritis [26], Parkinson’s disease [27], hyperuricemia, nonalcoholic steatohepatitis [28], and various cancers [29] such as ovarian cancer [30], melanoma [31], and GBM [32,33,34].

Normal regulation of the cell cycle progression relies on the involvement and coordination of multiple transcription factors. An abnormal cell cycle significantly promotes the rapid multiplication of cancerous cells [35]. Triggering cell cycle arrest may inhibit cancer cells growth by initiating the apoptotic process or suppressing cell proliferation.

Building on our prior finding that TB exerts potent anti-proliferative effects in glioma via cell cycle modulation [20], we hypothesized that small molecules mimicking its transcriptional signature could replicate its therapeutic benefits while offering superior druggability. To test this, we first utilized the CMAP to identify candidate drugs that mimic TB’s effects on gene expression related to glioma cell cycle regulation. Using the TB-induced gene expression profile as a query signature, we systematically screened for compounds with high connectivity scores in pathways related to cell cycle arrest and tumor suppression. This unbiased approach enabled the identification of structurally simple, physicochemical candidates with potential CNS activity. Among these, Methylstat emerged as a top candidate based on its high connectivity score, favorable physicochemical properties, and predicted mechanism of action. As a selective inhibitor of Jumonji C (JmjC) domain-containing demethylases, methylstat is closely connected to the vital roles of JmjC enzymes, which play critical roles in a wide range of physiological and pathological conditions, including cancer, neurological diseases, inflammation, developmental disorders and metabolic disturbances [36,37,38,39]. Then, we conducted in vitro experiments to evaluate the effects of methylstat on cell viability, proliferation, cell cycle distribution, and the expression of relevant molecular markers in human glioma cell lines. U251 cells (glioblastoma lineage) and HOG cells (oligodendroglioma lineage) were deliberately selected based on their well-characterized molecular profiles and established utility as representative models, which allows for a comparative evaluation of compound efficacy across distinct glioma subtypes. Additionally, we constructed an in vitro BBB model to assess the ability of methylstat to penetrate the BBB, a crucial factor for their effectiveness in treating glioma in the CNS. Thus, our study integrates a systems biology approach with functional experimental validation to bridge the gap between natural product pharmacology and translational oncology.

## 2. Results

### 2.1. Transcriptome Sequencing Analysis and Connectivity Map Drug Screening in Human Glioma Cells

**Figure 1:**
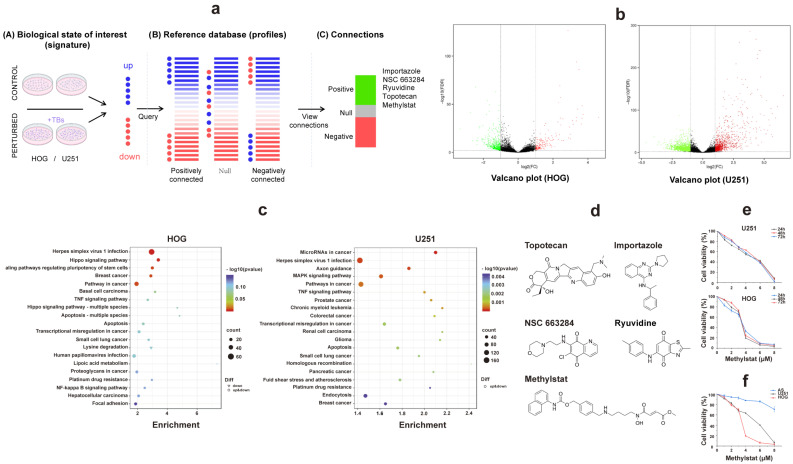
Comprehensive Analysis of Transcriptome Sequencing and Connectivity Map in Human Glioma Cells. (**a**) Network analysis of gene co-expression patterns in TB-treated HOG and U251 glioma cells using the Connectivity Map database. Five small-molecule compounds showing significant positive correlations are identified; (**b**) Volcano plots depicting differential gene expression (DEGs) in TB-treated glioma cells. Black: genes that are not significantly deregulated; green: downregulated genes; red, upregulated genes; (**c**) KEGG enrichment analysis of differentially expressed genes in HOG and U251 cells. Bubble size corresponds to the number of enriched genes per pathway, while color intensity represents statistical significance (−log_10_ (*p*-value)); (**d**) The chemical structures of topotecan, importazole, NSC-663284, methylstat, and ryuvidine; (**e**) Cell viability assessment by CCK-8 assay in glioma cells treated with methylstat (0–6 μM for U251, 0–4 μM for HOG) for 24, 48, and 72 h. (**f**) Differential sensitivity to methylstat exposure (48-h treatment) across glioma cell lines (HOG, U251) versus primary cultured astrocytes. Values are presented as mean ± SD (*n* = 3).

Building upon previous studies [20] that investigated the 50% inhibitory concentration (IC_50_) values of TB in human glioma cells, we extracted total RNA from HOG and U251 glioma cell lines treated with TB at concentrations of 150 μg/mL and 200 μg/mL, respectively, for a duration of 4 h. Comprehensive transcriptome sequencing was subsequently performed to characterize the global gene expression profiles (Figure 1a,b). Quality assessment of the sequencing data revealed strong correlations in gene expression levels across biological replicates, demonstrating high reproducibility and confirming the reliability of the dataset. The raw sequence data have been deposited in the Genome Sequence Archive (GSA-Human: HRA011952) at the National Genomics Data Center (NGDC), China National Center for Bioinformation, and are publicly available at https://ngdc.cncb.ac.cn/gsa-human (accessed on 19 June 2025) [40,41].

Our in-depth analysis focused on identifying differentially expressed genes (DEGs) (listed in Tables S1 and S2). To visualize the differential expression patterns, we employed an intuitive volcano plot (Figure 1b). Under stringent screening criteria, we identified 3419 DEGs in U251 cells, of which 1260 were downregulated and 2159 were upregulated (Figure 1b). In HOG cells, we detected 705 DEGs, with 172 downregulated and 533 upregulated (Figure 1b). Gene Ontology (GO) and Kyoto Encyclopedia of Genes and Genomes (KEGG) enrichment analyses revealed that these DEGs play pivotal roles in cellular regulation, metabolism, signal transduction, and cancer-related pathways. Specifically, they are involved in the Hippo, ERK/MAPK, and tumor necrosis factor (TNF) signaling pathways, which are directly associated with glioma biology (Figure 1c). Previous studies have shown that the Hippo-YAP signaling pathway can inhibit glioma cell growth in vitro and in vivo by inducing cell cycle arrest [42]. Additionally, upregulation of the ERK/MAPK signaling pathway has been implicated in the expansion of mitogenic stimuli and the promotion of cell proliferation in malignant gliomas [43].

Then, the CMAP tool was used to identify potential therapeutic small molecule compounds (Figure 1a,d). Notably, methylstat exhibits selective targeting of JMJD2 enzymes, effectively suppressing tumor cell proliferation [39]. This effect is particularly notable in multiple myeloma, where it halts the cell cycle at the S phase [44]. Given the aberrant expression of JMJD2A and its involvement in the Akt-mTOR pathway, which aligns with our research focus on the effects of TB on glioma cell cycle regulation, we aimed to intensively study methylstat for its potential as a glioma therapeutic. This approach embodies the principles of precision medicine and offers hope for innovative treatments.

To investigate the therapeutic potential of methylstat, we treated HOG and U251 glioma cells, as well as control primary cultured astrocytes, with varying concentrations (0–8 μM) of methylstat. Cell viability was measured using a Cell Counting Kit-8 (CCK-8) assay at different time points (24, 48, and 72 h). The results demonstrated that methylstat dramatically inhibited glioma cells’ proliferation in a dose-dependent manner (Figure 1e). Extending the treatment duration to 48 and 72 h did not markedly alter the IC_50_ values of U251 and HOG cells, indicating that the drug’s inhibitory effect on glioma growth was not time-dependent. We observed slight differences in sensitivity to methylstat among brain glioma cell lines from different sources, with HOG cells exhibiting greater sensitivity. Importantly, the survival rate of primary cultured astrocyte was 70.78 ± 5.16% at a high methylstat concentration of 8 μM for 48 h, suggesting that the drug is less toxic to normal cells. In contrast, under the same treatment conditions, the survival rates of HOG and U251 glioma cells were significantly reduced to 3.22 ± 1.85% and 6.49 ± 3.38%, respectively (Figure 1f). These data underscore the selective inhibitory effect of methylstat on glioma cells while sparing normal astrocytes. In subsequent tests, 4 μM and 6 μM were selected as high effective doses of methylstat for HOG and U251 cells, respectively. Cell samples were collected 48 h after dosing for subsequent assays.

### 2.2. Methylstat Does Not Induce Glioma Apoptosis

**Figure 2:**
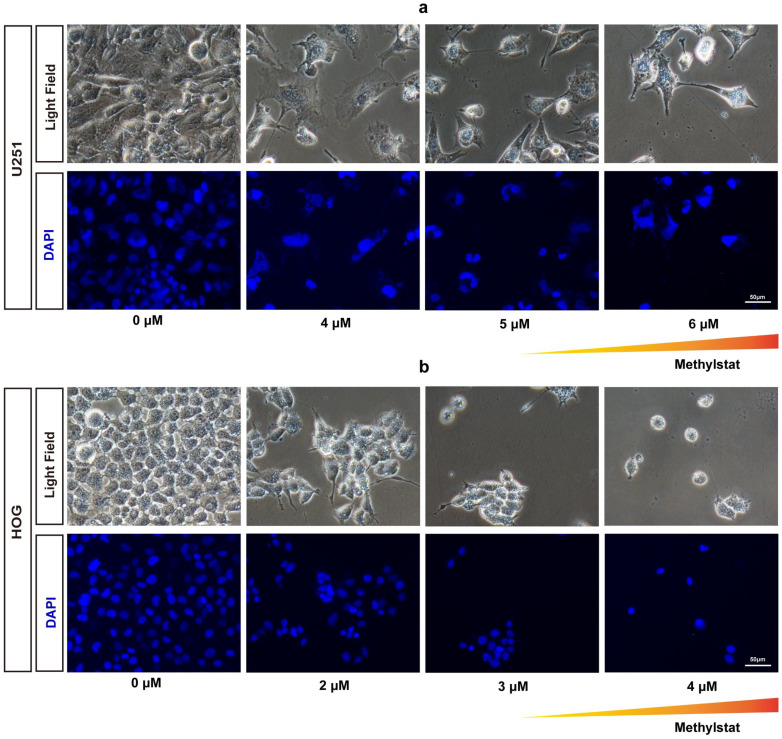
Morphological Changes and Nuclear Staining in Methylstat-Treated Glioma Cells. (**a**) Morphological changes and nuclear staining (DAPI) of U251 glioma cells after treatment with varying concentrations (0–6 μM) of methylstat for 48 h. The triangular gradient overlay indicates the methylstat concentration gradient (0 μM to 6 μM); (**b**) Morphological changes and DAPI of HOG glioma cells treated with varying concentrations (0–4 μM) of methylstat for 48 h. The triangular gradient overlay indicates the methylstat concentration gradient (0 μM to 4 μM);. Scale bar: 50 μm.

To investigate the effects of methylstat on HOG and U251 glioma cells, we conducted fluorescent staining to observe cell morphology after 48 h of methylstat treatment. Compared to the control group, the morphology of the cells in the experimental group underwent significantly changes, and the number of glioma cells was notably reduced. Specifically, methylstat-treated U251 cells exhibited a shrunken morphology with nuclear fragmentation (Figure 2a), while the nuclei of HOG cells were significantly smaller than those in the untreated group (Figure 2b).

**Figure 3:**
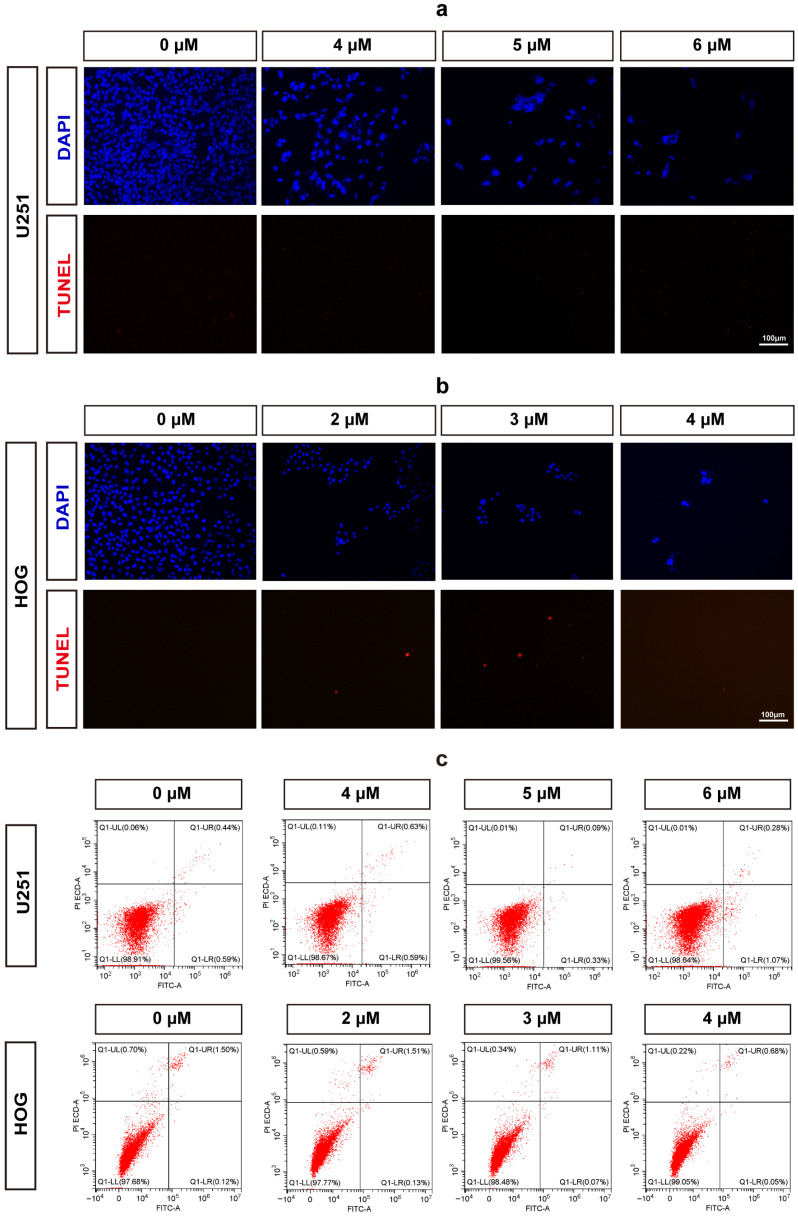
Assessment of Apoptosis in Methylstat-Treated Glioma Cells. (**a**) TUNEL/DAPI staining of U251 cells treated with methylstat (0–6 μM, 48 h); (**b**) TUNEL/DAPI staining of HOG cells treated with methylstat (0–4 μM, 48 h); (**c**) Annexin V/PI flow cytometry analysis of U251 (4–6 μM) and HOG (2–4 μM) cells demonstrates no statistically significant elevation in early (Annexin V+/PI−) or late (Annexin V+/PI+) apoptotic populations compared to untreated controls (mean ± SD, *n* = 3). Scale bar: 100 μm.

To further evaluate whether methylstat induces apoptosis in glioma cells, we performed terminal deoxynucleotidyl transferase dUTP nick-end labeling (TUNEL) and 4′,6′-diamidino-2-phenylindole (DAPI) staining assays. No positive signals indicative of apoptosis were observed in either U251or HOG cells at any of the concentrations tested following methylstat administration (Figure 3a,b). Additionally, we used Annexin V/PI double staining to detect apoptosis. The results showed no significant difference in the early and late apoptosis rates between the two glioma cell lines treated with high concentration of the methylstat and the control group (Figure 3c), further confirming that methylstat does not affect the apoptosis of glioma cells.

### 2.3. Methylstat Inhibits Glioma Cell Proliferation

**Figure 4:**
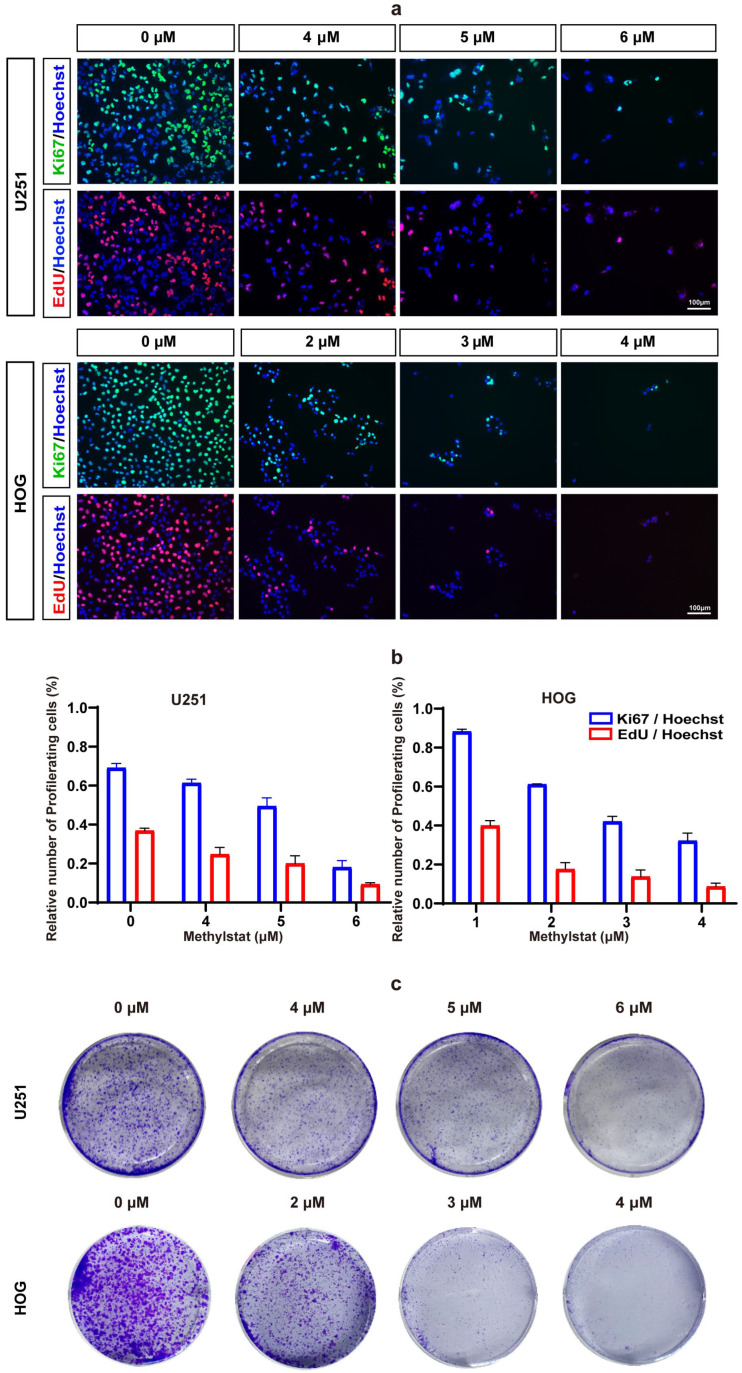
Methylstat Treatment Inhibit Glioma Cell Proliferation in vitro. (**a**) Ki67/EdU immunofluorescence staining in U251 and HOG cells treated with methylstat for 48 h. A significant reduction in Ki67-positive (green) and EdU-positive (red) cells indicates decreased proliferation; (**b**) Quantitative analysis reveals that methylstat treatment reduces the percentage of proliferating cells; (**c**) Colony formation assays (5000 cells/well seeded) show a dose-dependent decrease in colony counts following 10 days continuous methylstat treatment in both cell lines.

To further elucidate the impact of methylstat on glioma cells, we employed Ki67/EdU immunofluorescence staining. The results demonstrated that methylstat significantly inhibited glioma cells’ proliferation (Figure 4a). Following methylstat treatment, the percentage of Ki67-positive cells among the U251 cells decreased from 69.13 ± 2.27% to 18.12 ± 3.39%, while the percentage of EdU-positive cells decreased from 36.84 ± 1.26% to 9.39 ± 0.77% (Figure 4b). Similarly, among the HOG cells, the percentage of Ki67-positive cells decreased from 88.28 ± 1.17% to 32.19 ± 3.92%, and the percentage of EdU-positive cells decreased from 40.01 ± 2.50% to 8.68 ± 1.77% compared to the control (Figure 4b).

To further verify the effect of methylstat on glioma cell proliferation, we conducted colony formation assays. As anticipated, fewer colonies were formed in both U251 and HOG cells after 19 days of methylstat treatment (Figure 4c). These findings collectively indicate that methylstat can significantly inhibit the proliferation of glioma cells.

### 2.4. Methylstat Arrests the Cell Cycle at the G1/S and G2/M Phases in Glioma Cells

**Figure 5:**
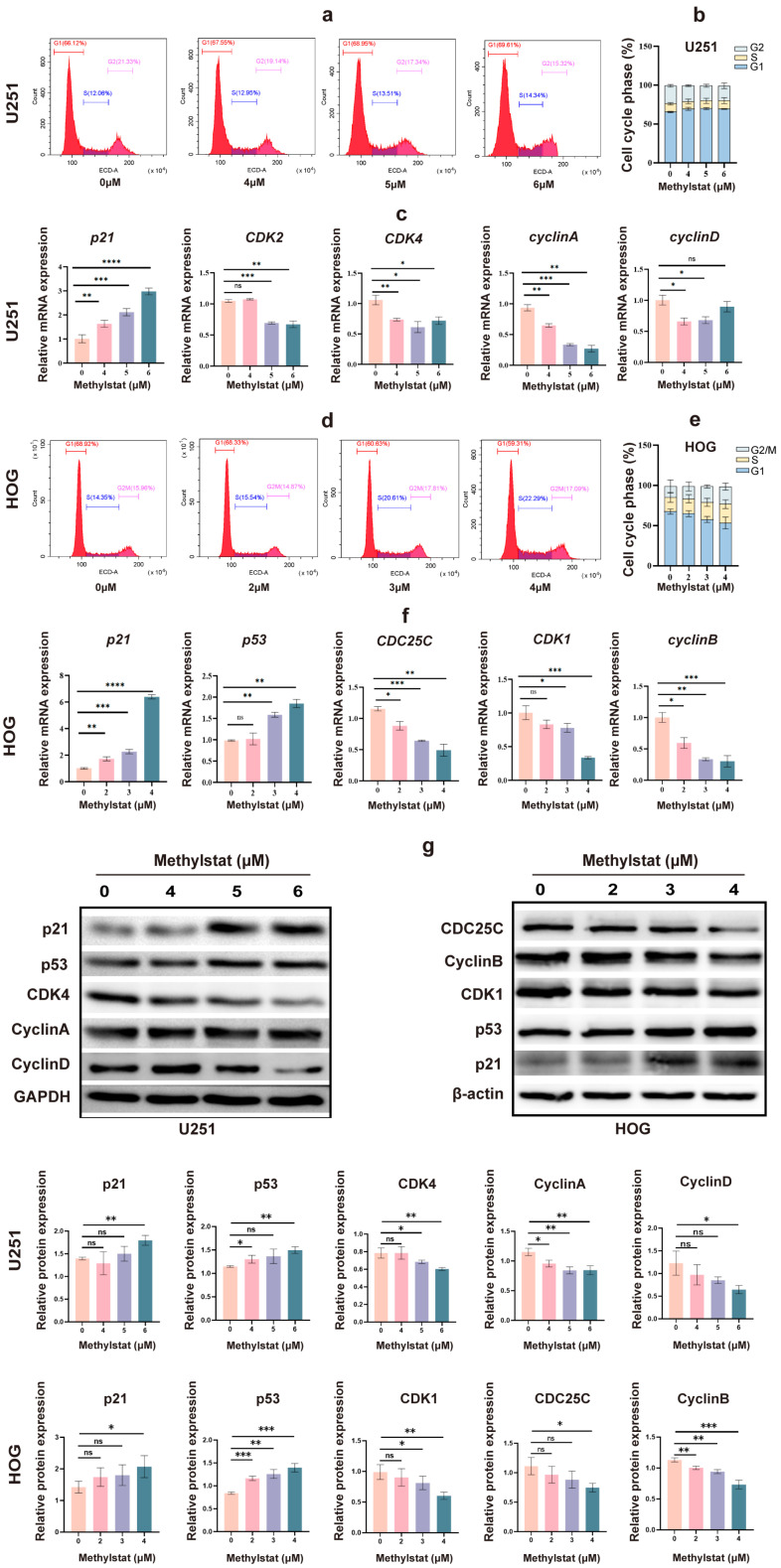
Effects of Methylstat on Cell Cycle Regulation in Glioma Cells. (**a**) Flow cytometry analysis of U251 cells treated with methylstat (0–6 μM, 48 h) reveals G1 phase accumulation. Red: G1 phase; Blue: S phase; Pink: G2 phase; (**b**) Quantitative assessment of cell cycle distribution in U251 cells after methylstat treatment; (**c**) qPCR analysis in U251 cells indicates downregulation of *cyclin D*, *cyclin A*, *CDK2*, and *CDK4*, with upregulation of p*21* upon methylstat treatment. Values are presented as mean ± SD of triplicate; (**d**) Flow cytometry of HOG cells treated with methylstat (0 to 4 μM, 48 h) shows an increase in G2 phase arrest. Red: G1 phase; Blue: S phase; Pink: G2/M phase; (**e**) Quantitative assessment of cell cycle distribution in HOG cells after methylstat treatment; (**f**) In HOG cells, qPCR shows reduced expression of *cyclin B*, *CDK1*, and *CDC25C*, but enhanced *p53* and *p21* levels post-treatment. Values are presented as mean ± SD of triplicate; (**g**) Western blot analyses of the expression levels of p21, p53, CDK4, cyclin A, cyclin D, CDK1, CDC25C, Cyclin B from U251 cells or HOG cells treated with methylstat, where GAPDH and β-actin were used as loading controls for U251 and HOG cells, respectively. Statistical significance: * *p* < 0.05, ** *p* < 0.01, *** *p* < 0.001, **** *p* < 0.0001; ns, not significant (Student’s *t*-test).

To further investigate the effects of methylstat on the cell cycle in HOG and U251 cells, we incubated the cells with methylstat and subsequently stained them for cell cycle distribution analysis using flow cytometry. In U251 cells, methylstat treatment led to an accumulation of cells in the G1 phase of the cell cycle (Figure 5a). Specifically, at a concentration of 6 μM methylstat, the proportion of U251 cells in the G1 phase increased from 65.38 ± 0.93% to 69.38 ± 0.47%, while the proportions of cells in the G2 phase decreased from 23.20 ± 1.65% to 18.92 ± 3.70% compared to the control (Figure 5b). Quantitative polymerase chain reaction (qPCR) analysis revealed dose-dependent suppression of key G1/S transition regulators by methylstat. At a concentration of 6 μM, expression levels were significantly reduced for *CDK2* (0.64-fold, *p* < 0.01), *CDK4* (0.68-fold, *p* < 0.05), and *cyclin A* (0.29-fold, *p* < 0.01). *Cyclin D* exhibited a biphasic response, showing progressive suppression at lower concentrations (0.66-fold at 4 μM, *p* < 0.05; 0.68-fold at 5 μM, *p* < 0.05), but stabilized at 6 μM (0.89-fold, not statistically significant). Notably, the cyclin-dependent kinase inhibitor *p21* was strongly upregulated (2.95-fold, *p* < 0.0001) under the same conditions (Figure 5c). Interestingly, HOG cells treated with methylstat exhibited cell cycle arrest in the G2 phase (Figure 5d). Compared to the control group, the proportion of cells in the G2 phase increased from 14.12 ± 7.81% to 21.84 ± 4.34%, while the proportion of cells in the G1 phase decreased from 67.52 ± 3.23% to 53.28 ± 7.36% (Figure 5e). qPCR analysis of G2-M phase-associated genes, including *cyclin B*, *CDK1*, and *CDC25C*, showed decreased expression of key G2-phase regulatory proteins following methylstat treatment. Concurrently, this molecular modulation was accompanied by a dose-dependent (4 μM) induction of *p53* (1.88-fold, *p* < 0.01) and *p21* (6.39-fold, *p* < 0.0001), key mediators of cell cycle arrest (Figure 5f).

Western blot analysis further corroborated the above findings. In U251 cells, it showed decreased expression of CDK4, Cyclin A, and Cyclin D, accompanied by an increase in the protein levels of p21 and p53, with glyceraldehyde 3-phosphate dehydrogenase (GAPDH) used as a loading control (Figure 5g). In HOG cells, Western blot analysis demonstrated downregulation of CDK1, CDC25C, and Cyclin B, coupled with an upregulation of p53 and p21, utilizing β-actin as the loading control (Figure 5g). Collectively, these findings indicate that methylstat can induce cell cycle arrest in glioma cells in a cell-line specific manner, with U251 cells arrested in the G1 phase and HOG cells arrested in the G2 phase.

### 2.5. Methylstat Reduces the Expression Level of JMJD2A and Inhibits JMJD2A Enzymatic Activity in Glioma Cells

**Figure 6:**
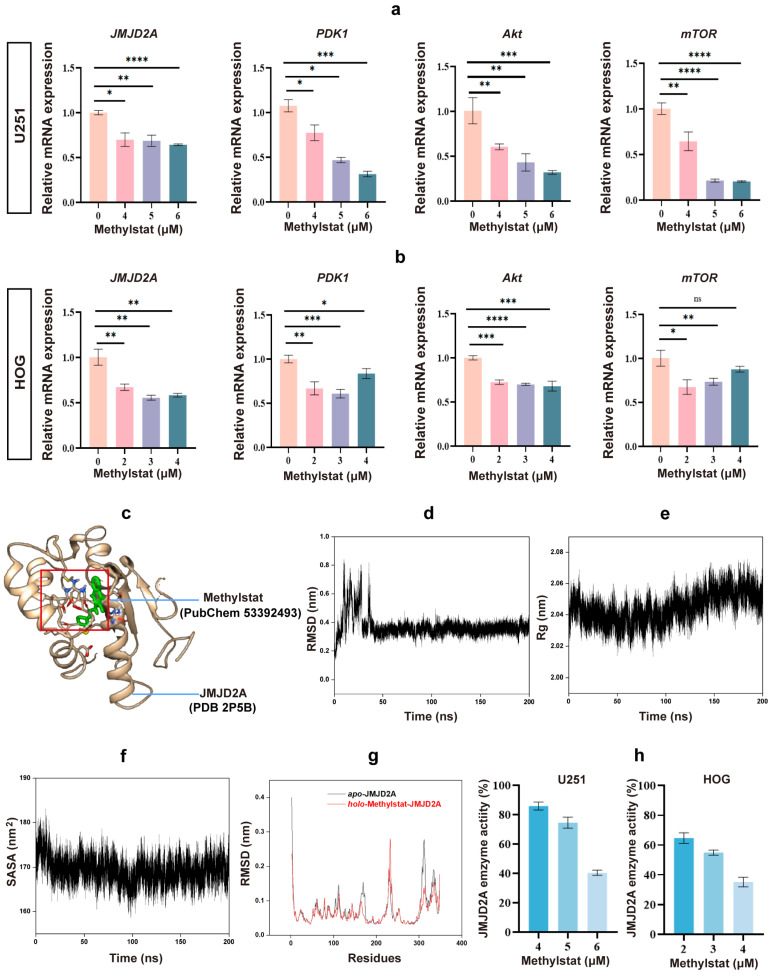
Effects of Methylstat on JMJD2A Expression and Enzymatic Activity in Glioma Cells. (**a**) qPCR analysis of U251 cells treated with methylstat (0–6 μM, 48 h) shows significant reductions in *JMJD2A*, *PDK1*, *AKT*, and *mTOR* mRNA levels. Values are presented as mean ± SD of triplicate experiments; (**b**) qPCR analysis of HOG cells treated with methylstat (0–4 μM, 48 h) reveals a marked decrease in *JMJD2A*, *PDK1*, *AKT*, and *mTOR* mRNA levels. Values are presented as mean ± SD of triplicate experiments; (**c**) 3D structure modeling, based on the JMJD2A crystal structure (PDB 2P5B) and Methylstat (PubChem 53392493), reveals stable binding of methylstat within the active site of JMJD2A; (**d**) Root mean square deviation (RMSD) profile of the JMJD2A–methylstat complex over 200 ns of molecular dynamics (MD) simulation; (**e**) Radius of gyration (Rg) trajectory during the MD simulation, reflecting structural compactness; (**f**) Solvent-accessible surface area (SASA) of the complex throughout the simulation, indicating conformational stability; (**g**) Root mean square fluctuation (RMSF) analysis comparing backbone flexibility of JMJD2A in apo and holo states; (**h**) Effect of methylstat on JMJD2A enzyme activity of glioma cells. Statistical significance: * *p* < 0.05, ** *p* < 0.01, *** *p* < 0.001, **** *p* < 0.0001; ns, not significant (Student’s *t*-test).

Methylstat is known to selectively inhibit JmjC domain-containing histone demethylases in cells [38,39]. Among these, JMJD2A is the most extensively studied member of the JMJD2 family, which contains the JmjC domain [45,46,47]. Previous evidence suggests that JMJD2A demethylates H3K9 and H3K36, thereby activating PDK1 expression and subsequently stimulating the Akt-mTOR pathway to promote glioma growth [47,48]. In this study, we observed a significant decrease in *JMJD2A* levels, as well as notably lower levels of *PDK1*, *AKT*, and *mTOR*, in methylstat-treated U251 and HOG cells compared to the untreated group (Figure 6a,b).

To elucidate the molecular mechanism underlying methylstat’s inhibitory effect, 3D structure modeling of the JMJD2A–methylstat complex was performed using the JMJD2A crystal structure (PDB 2P5B) and the chemical structure of methylstat (PubChem 53392493) [48,49,50]. The results revealed that methylstat is specifically bound within the catalytic pocket of JMJD2A through crucial interactions with residues Glu478, Arg482, and Trp550 (Figure 6c). Notably, the benzene ring of methylstat aligns parallel to the indole ring of Trp550, facilitating strong π–π stacking interactions. These interactions effectively occupy the active site and are likely to block substrate access, suggesting a direct mechanism for enzymatic inhibition.

To further evaluate the dynamic stability of the JMJD2A–methylstat complex, 200 ns molecular dynamics (MD) simulations were performed [51,52]. Key parameters including root mean square deviation (RMSD), radius of gyration (Rg), and solvent-accessible surface area (SASA) were analyzed throughout the simulation. The RMSD reached equilibrium by approximately 40 ns and remained stable, with an average value of 0.35 ± 0.03 nm (Figure 6d), indicating minimal conformational changes in the complex. The Rg exhibited an average value of 2.04 ± 0.01 nm (Figure 6e), reflecting a compact and stable structure. Moreover, the SASA, a crucial determinant of protein folding and stability, remained relatively constant at 168.75 ± 2.44 nm^2^ (Figure 6f), further supporting the structural integrity of the complex. Binding free energy (ΔG) calculations using gmx_MMPBSA in generalized Born (GB) mode, based on the final 20 ns of the trajectory, yielded a favorable ΔG of −36.1 ± 3.1 kJ/mol (Table 1), confirming a strong and thermodynamically stable interaction between methylstat and JMJD2A. Additionally, root mean square fluctuation (RMSF) analysis of the JMJD2A backbone in both apo and holo states revealed reduced flexibility at the binding site residues, highlighting a stable and well-defined interaction interface (Figure 6g). Consequently, the MD simulations demonstrated that Methylstat exhibits high affinity and stable binding to the active site of JMJD2A.

To confirm methylstat’s inhibitory effect on JMJD2A in glioma cells, we assessed the enzyme’s activity using a JMJD2A histone demethylase kit. As anticipated, the results demonstrated that methylstat effectively inhibited the enzymatic activity of JMJD2A in glioma cell lines (Figure 6h). The rates of enzyme activity inhibition were comparable between the two cells following high concentration treatment. However, the inhibition rate of enzyme activity at the IC_50_ concentration was significantly higher in HOG cells than in U251 cells, consistent with our previous findings that HOG cells exhibit greater sensitive to Methylstat compared to U251 cells. Collectively, these results indicated that methylstat treatment of glioma cells reduces the expression level of JMJD2A and inhibited its enzymatic activity, thereby providing a potential mechanism for its anti-glioma effects.

The calculation of ΔG for the methylstat–JMJD2 complex was conducted using gmx_MMPBSA in generalized Born (GB) mode, based on 2000 snapshots extracted from MD simulations trajectories spanning 180 to 200 ns. ΔGGAS = ΔEEL + ΔVDW. ΔGSOLV = ΔEGB + ΔESURF. ΔG = ΔGGAS + ΔGSOLV. All energy values were expressed in kcal/mol.

### 2.6. Methylstat Can Penetrate the BBB Model

**Figure 7:**
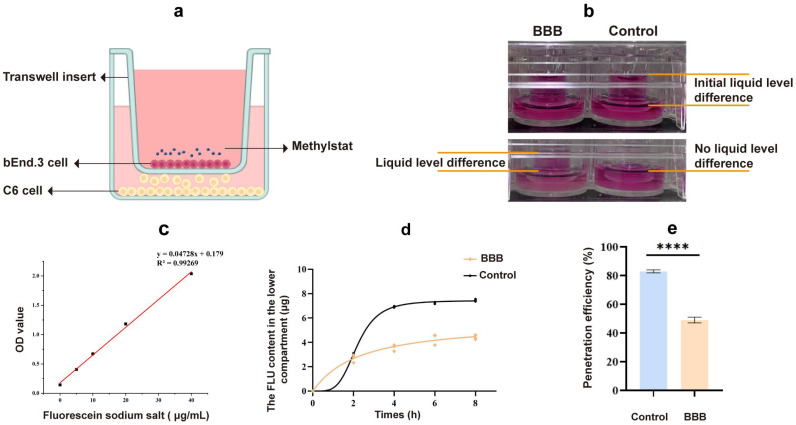
Transport of drugs across the in vitro BBB model. (**a**) Illustration of the in vitro BBB model; (**b**) A 4 h leak test; (**c**) Standard curve for sodium fluorescein quantification; (**d**) Fluorescein sodium standard curve; (**d**) Fluorescein sodium content in the bottom chambers at different time periods; (**e**) Fluorescein sodium permeability rate after 8 h. Statistical significance: **** *p* < 0.0001.

To investigate the ability of methylstat to penetrate the BBB, we first evaluated an in vivo BBB model co-cultured with mouse brain endothelial cells (bEnd.3) and rat glioma cells (C6) (Figure 7a), following protocols refined from previously published studies [53,54]. After a 96-h period of co-culture, a 4 h leak test was conducted. Compared to the control group without cell inoculation, the experimental model group maintained a notable difference in liquid levels between the upper and lower chambers (Figure 7b). In contrast, the liquid levels in the lower chamber of the control group were flush, and the liquid level difference disappeared, suggesting that the cells seeded at this time formed a dense and tightly bond layer.

In the in vitro BBB model, the quality control standard dictates that the permeability of hydrophilic substances should be controlled to a certain extent. The permeability coefficient of sodium fluorescein (NaFl) can serve as one of the indicators of the success of establishing a barrier model. We drew the NaFl standard curve within the concentration range of 0–40 μg/mL (Figure 7c). It was observed that the NaFl concentration had a linear relationship with the absorbance within this range, allowing the NaFl concentration to be calculated from the absorbance. Following an 8 h period, there was a significant decrease in the permeability of the model group compared to the blank control group (Figure 7d). The NaFl permeability rate was 48.94% in the model group and 82.9% in the blank group (Figure 7e), indicating that the model presented a barrier to small molecule substances. The permeability coefficient of the cell barrier model we established for NaFl was 4.17 (±0.31) × 10^−6^ cm/s, which is consistent with previous literature reports [55,56], suggesting that the cell barrier we established meets the experimental requirements. Using the in vitro BBB model, our test results show that methylstat can pass through the BBB with a pass rate of 13.07 ± 0.23%.

## 3. Discussion

GBM is a highly aggressive and life-threatening primary brain tumor. These characteristics are largely attributable to its inherent resistance to a broad spectrum of therapeutic strategies. Despite extensive research efforts, GBM persists as one of the most prevalent and formidable primary malignant brain tumors, with patients typically having a median survival of less than 15 months [5]. The inherent invasiveness of GBM makes complete surgical excision challenging, and these tumors frequently exhibit resistance to chemotherapy and reduced sensitivity to radiation, accompanied by significant adverse effects [3]. Alarmingly, for Grade IV glioblastoma multiforme, the most aggressive form, the 5-year survival rates are estimated to be a mere 4–5%, imposing a substantial financial burden on both patients and society [5]. Over the past few years, novel GBM therapies have emerged, including gamma knife, proton beam, tumor-treating fields, and inhibitors targeting epidermal growth factor receptor (EGFR), vascular endothelial growth factor (VEGF), receptor tyrosine kinases (RTKs), and the PI3K pathway [6,7,8,9,10,11,12]. Immunotherapeutic approaches, such as chimeric antigen receptor (CAR)-T/NK cells [13,14], dendritic cell vaccines [15], and checkpoint inhibitors [16], are also under development. However, a major challenge persists in achieving targeted delivery across the BBB, and many of these approaches provide only limited benefit compared to standard treatments. Consequently, the pursuit of effective novel therapeutics is of paramount importance.

Our previous research confirmed the inhibitory potential of TB, a theaflavin derivative, on glioblastoma via caspase-3-dependent pathways and PTEN/AKT signaling pathway, ultimately leading to cell cycle arrest and proliferation suppression [20]. However, the heterogeneity of TB, which encompasses carbohydrates, proteins, and flavonoids, coupled with the potential use of toxic solvents during purification, necessitates the identification of a more suitable candidate for clinical application [57,58]. To address these limitations, we examined the expression profile of TB-treated HOG and U251 cells compared to untreated cells using whole-gene microarray analysis and observed significant changes in the gene expression profile of the treated glioma cells. By setting the criterion for differential expression (fold change ≥ 2, *q*-value <0.05), we identified differential genes between treatment and control groups. GO enrichment analysis revealed changes in gene expression related to membrane and organelle function, with enrichment in signaling activity. KEGG pathway analysis indicated that the differential genes were associated with Hippo, MAPK, and TNF signaling pathways, which are essential for the cell cycle. This was consistent with previous studies where TB-targeted cell cycle arrest inhibited glioma cell growth [20]. It has been documented that activating Hippo can phosphorylate and inactivate the transcriptional coactivator YAP/TAZ through the MST kinase/LATS kinase axis, thereby inhibiting tissue growth and cell proliferation [59]. The MAPK signaling pathway is a cascade of phosphorylation processes in which hyperactivation of the key kinase protein ERK1/2 induces cell cycle arrest by inducing the accumulation of cell cycle-dependent kinase inhibitors such as p21 [60]. TNF promotes the expression of the *c-myc* gene, thereby facilitating the transition of cells from the G0 phase to the G1 phase of the cell cycle. Additionally, TNF-α impairs the S-G2/M cell cycle checkpoint in HaCaT cells (human immortalized epidermal cells) by inhibiting the PI3K-Akt signaling pathway [61].

The CMAP platform ranks compounds by connectivity scores (−1 to 1) that quantify transcriptional similarity to the query (TB treatment), prioritizing candidates with positive scores suggesting shared/synergistic effects and reproducible high-magnitude associations. Through this analysis, we identified five drug candidates with the highest positive connectivity scores (mean score ≥ 0.5) (Figure 1). Among them, importazole, which restrains the nuclear transport receptor importin-β, stands out; however, the precise function of importin-β across the cell cycle remains poorly understood. Meanwhile, the roles of ryuvidine and topotecan, ranking third and fourth, respectively, in glioma therapy have been extensively investigated. Topotecan, which is currently being evaluated as an adjuvant therapy for malignant glioma, has shown promising results in early clinical trials. Sustained convection-enhanced delivery of topotecan has demonstrated therapeutic potential in glioma treatment, with observed tumor improvement in treated patients [62]. NSC-663284 is recognized as a CDC25 dual-specificity phosphatase inhibitor, capable of suppressing the activities of all three CDC25 phosphatases and reducing CDC25 protein expression, thus hindering the activation of downstream CDKs and inducing cell cycle arrest [63,64,65,66]. These outcomes validate the reliability of our CMAP-based drug screening methodology. Methylstat emerged as a promising small-molecule drug. These compounds, distinguished by their well-defined structures and ease of purification, offer advantages for in vivo pharmacological mechanism investigations, notably due to their enhanced ability to cross the BBB. In this study, methylstat acted as a histone demethylase inhibitor, targeting enzymes like JMJD2A. In vitro studies have shown that this drug exerts anti-proliferative effects on primary myeloma cells, arresting the cell cycle in the S phase [39,44]. It elevates the methylation levels of H3K27 in human umbilical vein endothelial cells (HUVECs), along with increasing p53 and p21 protein levels, ultimately inhibiting the expression of CyclinD1 [67]. Given our focus on identifying drugs that target the cell cycle, the small-molecule compound methylstat was selected for further in-depth investigation.

Our investigation demonstrated that methylstat substantially curbed the viability of HOG and U251 human glioma cell lines across a 0–8 μM concentration spectrum, with an escalating inhibitory effect as the dosage increased. Primary cultured astrocytes utilized as controls demonstrated significantly higher survival rates at 8 μM, highlighting methylstat’s selective toxicity towards cancerous cells over normal tissue. Microscopic analysis post-methylstat treatment revealed diminished cell counts, altered morphology, and reduced nuclear sizes in glioma cells relative to untreated counterparts (Figure 2). Apoptosis assays using TUNEL staining and Annexin V-FITC coupled with flow cytometry indicated no significant pro-apoptotic impact of methylstat on glioma cells (Figure 3). Subsequently, through the Ki67 staining results, the proportion of Ki67-positive cells has all decreased (Figure 4a,b). As a thymidine nucleoside analog, EdU is incorporated into the replicated DNA chain during the S phase of DNA synthesis and is an indicator of marking the S phase. With increasing concentrations of methylstat, the positive ratio of EdU decreased significantly, indicating that methylstat significantly inhibited the proliferative capacity of gliomas. In addition, in vitro colony formation assay found that the numbers of cell clones formed decreased and their size decreased after drug treatment, supporting the observation that methylstat can inhibit the proliferation of glioma cells (Figure 4c).

To elucidate the mechanism by which methylstat inhibits cell proliferation, we have conducted flow cytometry analyses on different glioma cell lines (U251 and HOG), revealing source-specific cell cycle arrest. In U251 cells derived from highly malignant astrocytes, Methylstat has induced an accumulation of cells in the G1 phase, evidenced by an upregulation of the tumor suppressor gene *p21* and subsequent inhibition of Cyclin–CDK complexes, notably Cyclin D–CDK4, characteristic of G1 arrest (Figure 5a–c,g). In contrast, HOG cells originating from oligodendroglial lineage with a lower malignancy were arrested at the G2 phase by methylstat, facilitated by an increase in p53 protein expression that enhanced p21 transcription and concurrently downregulated G2/M phase regulators such as cyclin B, CDK1, and CDC25C (Figure 5d–f,g), halting the progression into mitosis [68]. Methylstat’s mechanism of action involves the p53–p21–Cyclin B–CDK1 axis, where it inhibits Cyclin B–CDK1 complex activity and CDC25C phosphatase activity, effectively stalling cells in the G2 phase. Notably, HOG cells demonstrated significantly heightened sensitivity to methylstat compared with U251 cells, manifesting as more pronounced G2/M arrest and proliferation suppression at lower concentrations. This disparity probably stemmed from HOG cells retaining wild-type *p53*, which sustains functional p53 signaling pathways, whereas the U251 cells harbored mutant *p53*, disrupting cell cycle checkpoint regulation. These insights underscore the necessity of incorporating tumor lineage and genetic profiling into efficacy evaluations, highlight glioma heterogeneity, and reinforce the imperative for precision medicine in neuro-oncology, calling for future research to identify molecular biomarkers of drug sensitivity to refine clinical stratification strategies.

Moreover, methylstat served as a pivotal regulator of cell proliferation and metastasis, primarily by targeted modulation of the PI3K/AKT/mTOR pathway, which is crucial for cellular growth and malignant transformation [47]. Initiated by PI3K, this pathway generates phosphatidylinositol-3,4,5-trisphosphate (PIP3) to recruit PDK1 to the cell membrane, facilitating Akt activation. Our data show that Methylstat markedly decreases the mRNA expression levels of *PDK1*, *AKT*, and *mTOR* in glioma cells (Figure 6a,b), contributing to the suppression of the entire AKT/mTOR signaling cascade. Additionally, methylstat exerted a dual inhibitory effect on the histone demethylase JMJD2A, suppressing both its expression and enzymatic activity. This dual action further reinforces PDK1 downregulation, amplifies the overall pathway inhibition, and creates a well-coordinated feedback mechanism, thereby restraining glioma cell proliferation.

JMJD2A, a histone demethylase, plays a pivotal role in the progression of multiple cancer types. In lung and bladder cancers, its overexpression promotes the G1/S phase transition, thereby accelerating tumor cell proliferation [69]. JMJD2A overexpression is widespread, occurring in breast cancer [70], gastric cancer [71], prostate cancer [72], colorectal cancer [73], and nasopharyngeal carcinoma [46]. Recent studies also show its upregulation in glioma, where it drives tumor cell proliferation via Akt-mTOR pathway activation [47]. Consequently, these results firmly establish JMJD2A as an exceptionally promising therapeutic target spanning a wide array of cancers. In this study, we have demonstrated that methylstat, identified through a transcriptional signature-driven drug repurposing strategy, exerts robust anti-proliferative effects in glioma cells by specifically targeting JMJD2A. Utilizing AlphaFold2-based structural modeling, molecular docking, and comprehensive 200 ns MD simulations, we have revealed that methylstat forms a highly stable interaction with the catalytic JmjC domain of JMJD2A, primarily through persistent hydrogen bonds with key active-site residues (Glu478, Arg482, and Trp550) and strong π–π stacking between the benzene ring of Methylstat and the indole ring of Trp550 (Figure 6c). These interactions effectively occupy the substrate-binding pocket, as evidenced by minimal conformational fluctuations (RMSD = 0.35 ± 0.03 nm), compact structural integrity (Rg = 2.04 ± 0.01 nm), and consistent solvent accessibility (SASA = 168.75 ± 2.44 nm^2^) throughout the simulation. The favorable binding free energy (ΔG = −36.1 ± 3.1 kJ/mol) further supports a thermodynamically stable interaction (Figure 6d–g). Experimental evidence has demonstrated that methylstat forms a high-affinity interaction with the active site of JMJD2A, significantly reducing its enzymatic activity to 40.41 ± 1.76% in U251 cells and 35.05 ± 3.24% in HOG cells under high-dose treatment relative to controls (Figure 6h). These results correlate with the observed dose-dependent inhibition of JMJD2A activity and the greater sensitivity of HOG cells, which is probably due to higher JMJD2A expression levels.

Effective glioma treatment depends on the ability of therapeutic drugs to successfully traverse the BBB. The BBB, as a key physiological barrier, is composed of brain capillary walls (endothelium, basement membrane) and glial cells, which separate the CNS from harmful substances in the blood [74]. The strict control of the paracellular diffusion by BBB and the regulation of the polar molecular permeability ensure the safety of the CNS [75]. Although data from in vivo animal model studies are more direct and reliable than those from in vitro studies, the use of in vivo animal models in determining injury mechanisms and drug testing is limited due to species differences at partial genomic and molecular levels [76]. In contrast, in vitro BBB models provide cost-effective and high-throughput alternatives to study the mechanism of drug transport through the BBB, especially with human cell lines, to reduce the impact of interspecific differences [77,78] Our simplified in vitro BBB model, combining bEend.3 and C6 cell lines, showed tight cell-to-cell binding after a 4 h permeability test. The integrity of the model was verified by a NaFl permeability test, with significantly reduced permeability compared to the apparent permeability coefficient of 4.17 (± 0.31) 10^−6^ cm/s, which is consistent with the available literature [55,56]. High-performance liquid chromatography (HPLC) analysis showed that methylstat can cross the BBB. After 4h, methylstat penetration was 4.434%, thereby establishing its effective BBB-penetrating ability (Figure 7). Taken together, with its high-affinity inhibitory effect on JMJD2A and robust anti-proliferative activity against glioma cells factored in, these findings strongly highlight methylstat’s significant potential as a promising therapeutic agent for glioma treatment.

## 4. Materials and Methods

### 4.1. Cell Lines and Cell Culture

Human glioma cell lines U251 and HOG were obtained from the American Type Culture Collection (ATCC) and the Feng Lab, Emory University respectively. Primary astrocytes were isolated from the cortex of newborn mice. The mouse brain endothelial cells line bEnd.3 and the rat glioma cell line C6 were prchased from HyCyte Biology (Suzhou, China). All cell lines were cultured in Dulbecco’s Modified Eagle Medium (DMEM; Gbico, Grand Island, NY, USA) supplemented with 10% fetal bovine serum (FBS) (ExCell Bio, Shanghai, China) and 1% penicillin/streptomycin in a humidified incubator (Thermo Fisher Scientific, Waltham, MA, USA) with 5% CO_2_ at 37 °C. C6 cells were cultured in DMEM-F12 medium (Gbico, Grand Island, NY, USA) containing 2.5% FBS and 15% horse serum (HS; Solarbio, Beijing, China).

### 4.2. Drugs and Compound

Methylstat (cat: HY-1522, MCE, Shanghai, China) was dissolved in dimethyl sulfoxide (DMSO) (Sigma-Aldrich, St. Louis, MO, USA) to prepare a 20 mM stock solution, stored at −20 °C. Before each experiment, it was diluted with complete culture medium to produce the necessary concentration gradients. TB (cat: CN20131038721) was obtained from Hangzhou Minghe Biotechnology Co., Ltd. (Hangzhou, China).

### 4.3. Screening Differentially Expressed Genes and CMAP Analysis

To identify genome-wide differentially expressed genes, HOG and U251 cells were treated with 150 μg/mL and 200 μg/mL TB, respectively, for 4 h. Total RNA was then extracted using Trizol reagent. Based on the Sequencing By Synthesis (SBS) technology, the cDNA library was sequenced on the high-throughput sequencing platform of Biomarker Bio-tech (Qingdao, China). The assigned human genome (version: homo_sapiens.grch38_release.genom.fa) was used as a reference for sequence alignment and subsequent analysis.

DEGs between TB-treated cells and control cells were identified through DESeq2 analysis, in accordance with the established statistical and fold-change thresholds (fold change ≥ 2, *p*-value < 0.05). The gene expression signatures of TB-treated glioma cell lines, including the lists of significantly upregulated and downregulated genes, were used as input data for the Connectivity Map online web tool https://clue.io/ (accessed on 5 July 2022) [79]. Connectivity scores were calculated for each drug–glioma combination to investigate the correlation between drug and glioma characteristics. The CMAP platform utilizes a non-parametric, rank-based pattern-matching algorithm to generate scores ranging from −1 to +1. These scores quantify the transcriptional similarity or dissimilarity between the TB-induced gene expression signature and those of compounds in the CMAP database. A positive score suggests that the compound induces a gene expression pattern similar to TB treatment, while a negative score indicates a reversal of the disease-associated profile. In this research, drugs with notably positive scores (mean CMap score ≥ 0.5) were considered potential therapeutics, as they could replicate or amplify the transcriptional effects of TB. Based on this criterion, five drug candidates were selected and prioritized for further experimental evaluation.

### 4.4. Methylstat-JMJD2A: Molecular Docking and Dynamics Analysis

The interaction between JMJD2A and methylstat was studied using molecular simulation. The crystal structure of receptor JMJD2A was obtained from Protein Data Bank (accession ID:2P5B) with the of resolution of 1.99 Å [48] and the ligands, water molecules, heteroatoms, and any cocrystallized solvent were removed. Molecular docking between JMJD2A and methylstat was conducted using the AutoDock Vina 1.2.1 [49,50]. The grid box was located at the center of 20.138, 4.945, 16.383 and at dimensions of 24, 26, 24 points in x, y, and z directions with grid spacing of 1.0 Å. The parameters for exhaustiveness, energy range and number of modes were set as 300, 2.0 kcal/mol and 30, respectively. The conformation of the methylstat–JMJD2A complex that exhibited the lowest binding free energy was selected for further MD simulations. For MD simulations, the ff14SB forcefield [80] was applied to the JMJD2A, while the general AMBER force field (GAFF) [81] was utilized for the ligand methylstat. MD simulations of the methylstat–JMJD2A complex were conducted utilizing the GROMACS 2019.06 software for 200 ns. To evaluate the structural stability, the RMSD of heavy atoms of the ligand after least squares fitting to the backbone of JMJD2A, Rg, and SASA for the complex were calculated. RMSF analysis was conducted on the backbone of JMJD2A in apo and holo states. The ΔG of the two simulation systems were calculated using the gmx_MMPBSA method [51,52].

### 4.5. Cell Viability Assay

U251 and HOG cell lines were trypsinized and seeded into 96-well microplates at a density of 5 × 10^3^ cells/well for 24 h. The cells were then treated with different concentrations of methylstat for 24, 48, and 72 h, respectively. After each treatment period, the wells were washed once with incomplete cell culture media. Subsequently, 10 μL of CCK-8 reagents (Beyotime, Shanghai, China) and 100 μL of DMEM were added to each well, and incubated at 37 °C for one hour. The optical density (OD) was measured at 450 nm using a microplate reader. Cell survival rates were calculated using the appropriate formulae, and the IC_50_ of methylstat was determined using GraphPad Prism 7.0.

### 4.6. Cell Morphology and DAPI Staining

Methylstat-treated U251 and HOG cells were fixed with 4% paraformaldehyde (PFA) for 30 min at room temperature and then washed three times with phosphate-buffered saline (PBS). The cells were stained with DAPI for 10 min. The stained cells images were observed under a fluorescence microscope to examine the apoptotic nuclei.

### 4.7. Flow Cytometry for Cell Apoptosis and Cell Cycle Analysis

Cells were seeded in six-well plates at a density of 1.5 × 10^5^ cells/well for 24 h and then treated with different concentrations of Methylstat treatment for 48 h. Apoptosis was detected using an Apoptosis Detection Kit (Vazyme, Nanjing, China) according to the manufacturer’s instructions. The cells were incubated with Annexin V-FITC and propidium iodide (PI) Staining Solution (Yeasen, Shanghai, China) in the dark for 10 min. Apoptosis was measured and analyzed using a BD FACSAriaTM flow cytometer (Becton Dickinson, San Jose, CA, USA). For cell cycle analysis, the cells were stained using PI/RNase staining solution (cat: 550852, BD Biosciences, Woburn, MA, USA) at 37 °C for 20 min. After suspension, cell cycle distribution was analyzed using flow cytometry.

### 4.8. Colony Formation Assay

Approximately 5000 GBM cells per well were seeded in 6-well plates and cultured for 10 days. The colonies were fixed with 4% PFA and stained with crystal violet (Beyotime, Shanghai, China) for one hour. The colonies were then washed with PBS and the number of colonies was counted.

### 4.9. TUNEL Assay

HOG and U251 cells were cultured and seeded onto 24-well plates. After 48 h treatment with methylstat, cell apoptosis was evaluated using a TUNEL Kit (Vazyme, Nanjing, China), according to the manufacturer’s instructions. The cells were fixed with 4% PFA for 30 min at room temperature and incubated with 0.2% Triton-X 100 for 5 min. Then, the cells were incubated with TdT incubation solution at at 37 °C for 1.5 h. Finally, the cellular nuclei were stained with DAPI at room temperature for 10 min. The stained cells were visualized under a fluorescent microscope.

### 4.10. EdU and KI67 Assay

Different concentrations of methylstat were added to U251 and HOG cells for 48 h. Cell proliferation was evaluated using an EdU Cell Proliferation Kit with Alexa Fluor 555 (Epizyme, Shanghai, China) according to the manufacturer’s protocol. The cells were incubated with 20 μM of 5-ethynyl-20-deoxyuridine at 37 °C for 2 h and fixed with 4% PFA for 20 min at room temperature. The glioma cells were permeabilized in 0.3% Triton X-100 for 15 min and blocked with 5% goat serum for 30 min, followed by overnight incubation at 4 °C with the primary antibodies. The cells were then incubated with fluorescent second antibodies at room temperature for one hour. Afterward, Click reagent was added to the cells and incubated for 1 h at 37 °C in the dark. The nuclei were counterstained with Hoechst for 10 min. Images were captured using a fluorescence microscope, and the rates of Ki67 and EDU positive cells were calculated.

### 4.11. Real-Time PCR Analysis

After methylstat treatment, total RNA of cells was extracted using Trizol, and reverse transcription was performed using the HiScript III RT SuperMix for qPCR kit (Vazyme, Nanjing, China). Target genes were amplified with ChamQ Universal SYBR qPCR Master Mix (Vazyme, Nanjing, China) and measured using a CFX96™ Real-time System (Bio-Rad Laboratories, Hercules, CA, USA) according to the manufacturer’s instructions. Glyceraldehyde 3-phosphate dehydrogenase (GAPDH) was used as the reference gene, and the specific primers for the genes are listed in Table S3.

### 4.12. Western Blot Analysis

Following methylstat treatment, U251 and HOG cells were lysed with radioimmunoprecipitation assay (RIPA) buffer containing protease inhibitor for 30 min on ice. The cells were then centrifuged at 13,000 rpm for 30 min, and the protein concentration was measured using a Pierce BCA kit (Thermo Fisher Scientific, Waltham, MA, USA). Polyvinylidene difluoride membranes were blocked with 5% fat-free milk for 1 h, followed by overnight incubation at 4 °C with primary antibodies. The membranes were then incubated with peroxidase-conjugated goat anti-rabbit IgG for 1 h at room temperature and visualized using BeyoECL Star (Beyotime, Shanghai, China). Protein bands were detected using the ChemiDoc imaging system (Bio-Rad Laboratories, Hercules, CA, USA). Detailed information on the primary and secondary antibodies used is provided in Table S4.

### 4.13. In Vitro BBB Model Setup

C6 cells were seeded in 24-well plates at a density of 1 × 10^5^ cells/cm^2^ for 48 h. Then, bEnd.3 cells were cultured and seeded at a density of 2 × 10^4^ cells/cm^2^ onto the upper side of 6.5 mm Transwell^®^ polycar bonate membranes (0.4 μm pore size; Merck KGaA, Darmstadt, Germany), and the inserts were placed in 24-well plates filled with C6 cells. The plates were incubated, and the medium was replaced daily. Approximately 4–5 days later, a 4 h permeation test and a NaFl (Sigma-Aldrich, St. Louis, MO, USA) permeation test were conducted on the model after cell confluence to verify the successful establishment of the in vitro BBB model and ensure it met the standards for subsequent experiments.

### 4.14. Four-Hour Permeation Test

After 5 days of co-culture, the top part of the insert was replaced with 300 μL of DMEM medium, while the bottom of the 24-well plate was filled with 500 μL of C6 medium to maintain a consistent liquid level disparity. After 4 h incubation at 37 °C, no notable alteration in the liquid level disparity was observed compared with the control group, indicating close fusion of the cells.

### 4.15. In Vitro Permeability Assay

In vitro NaFl permeability was used to evaluate endothelial integrity. The endothelial permeability coefficients for fluorescein served as an indicator of a successful cell barrier model. NaFl was mixed into DMEM basic medium at varying concentrations of 0, 5, 10, 20 and 40 μg/mL, and its absorbance at 530 nm was measured to establish a standard curve. The culture medium was removed from the upper and bottom chambers and replaced with 300 μL of NaFl (20 μg/mL) diluted in DMEM and 500 μL of DMEM alone, respectively. After 4 h for NaFl treatment, 10 μL of the medium from the top chamber medium and 100 μL of the medium from the bottom chamber was sampled. The OD values was measured using a fluorescence microplate reader, and the transmembrane permeability coefficient was calculated using the previously established formula.

### 4.16. Drug Transport Through the In Vitro BBB Model

The co-cultured BBB model described above was used to test the permeability of methylstat. Into the Transwell^®^ top chamber, 100 μL of culture medium containing methylstat (3 μM and 5 μM) was added. At 1, 2, and 3 h, 10 μL of the culture solution in the lower chamber was collected, and 190 μL of acetonitrile was added for extraction. After centrifugation at 13,000 rpm for 10 min, the supernatant was collected. The supernatant was then analyzed by Agilent 1260 Infinity II HPLC system (Agilent Technologies, Santa Clara, CA, USA) to determine the amount of methylstat that had passed through the BBB.

### 4.17. Statistical Analysis

All data are presented as the mean ± standard deviation (SD) of at least three independent experiments performed. Statistical significance was evaluated by Student’s *t*-test. Statistically significant at * *p* < 0.05, ** *p* < 0.01, *** *p* < 0.001, and **** *p* < 0.0001 (n.s.: no significant difference).

## 5. Conclusions

**Figure 8:**
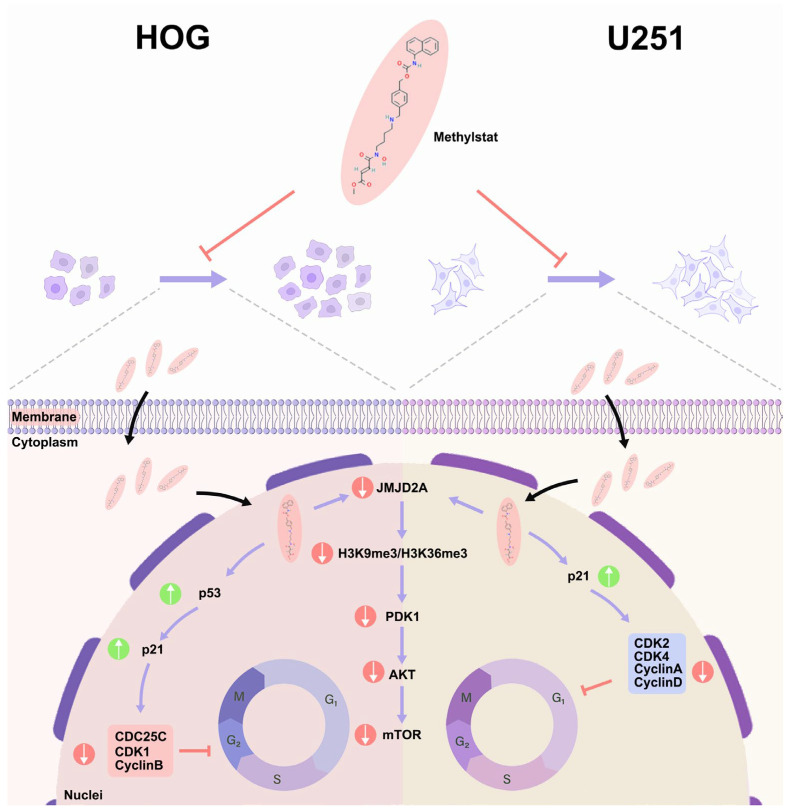
Schematic Diagram of Methylstat-Induced Cell Cycle Arrest and its Regulatory Mechanisms in Glioma Cells. Methylstat differentially regulates glioma cell cycle progression via two distinct pathways: in HOG cells, it induces G2/M arrest through a *p53*-dependent cascade (*p53* activation → *p21* upregulation → downregulation of *CDC25C*/*CDK1*/*cyclin B* → G2/M transition blockade), whereas in U251 cells, methylstat causes G1 accumulation via *p21*-mediated inhibition of the *cyclin D-CDK4* complex (p21 elevation → disruption of *cyclin D-CDK4* assembly → G1/S transition suppression); these differential effects are synergistically enhanced by methylstat-driven suppression of *JMJD2A* and attenuation of the PDK1/AKT/mTOR pro-proliferative signaling pathway, ultimately resulting in collective inhibition of glioma cell proliferation through dual-phase (G1 and G2/M) cell cycle arrest.

In conclusion, our study provides compelling evidence that methylstat, which was identified through the CMAP platform, exhibits potent anti-glioma effects. It accomplishes this by selectively targeting and inhibiting JMJD2A, a histone demethylase that plays a complex and crucial role in tumor proliferation across a diverse range of cancers. As illustrated in Figure 8, methylstat effectively controls the proliferation of glioma cells. It achieves this by initiating distinct cell cycle arrest processes. Specifically, it induces G1 phase arrest in U251 cells and G2/M phase arrest in HOG cells. The underlying mechanism of this regulatory action involves the activation of the p53-p21 signaling axis, which subsequently results in the downregulation of cyclin–CDK complexes. Importantly, throughout this process, normal astrocytes remain unaffected, underscoring the high degree of specificity that methylstat demonstrates. Through molecular docking and MD simulations, we have confirmed that methylstat forms a stable and specific bond with the catalytic site of JMJD2A. This binding not only suppresses the enzymatic activity of JMJD2A but also interrupts the PDK1-Akt-mTOR signaling pathway, a vital cascade that is essential for tumor growth and survival. A notable characteristic of methylstat is its significant BBB permeability. With a penetration rate of 13.07 ± 0.23%, it can effectively reach tumor sites within the brain. Furthermore, methylstat shows varying degrees of sensitivity among different glioma subtypes. HOG cells display a heightened responsiveness compared to U251 cells, indicating the potential for developing tailored therapeutic approaches. Overall, these findings collectively highlight the multifaceted pharmacological profile of Methylstat. It is characterized by its robust antiproliferative activity, precise cell cycle modulation, and excellent BBB penetrability, making it a promising candidate for glioblastoma therapy.

## Figures and Tables

**Table 1:** Binding free energy components for methylstat–JMJD2A MD systems using gmx_MMPBSA.

ΔEEL	ΔVDW	ΔEGB	ΔESURF	ΔGGAS	ΔGSOLV	ΔG	ΔEEL
−21.2 ± 5.7	−58.0 ± 2.7	51.1 ± 5.0	−8.0 ± 0.25	−79.2 ± 5.9	43.1 ± 4.9	−36.1 ± 3.1	−21.2 ± 5.7

## Data Availability

All the data supporting the findings of this work are available from the corresponding author upon request. The RNA-seq data for glioma cells treated with theabrownin are available at: https://download.cncb.ac.cn/gsa-human/HRA011952 (accessed on 2 September 2025).

## Supplementary Materials

The following supporting information can be downloaded at: https://www.mdpi.com/article/10.3390/ph18091344/s1, Figure S1: Western blot analysis in glioma cells treated with methylstat; Table S1. Differentially expressed genes identified in U251 cells treated with TB by RNA-seq.xlsx; Table S2. Differentially expressed genes identified in HOG cells treated with TB by RNA-seq.xls; Table S3. Primers used for qPCR in this study.xlsx; Table S4. Antibodies used for western blot in this study.xlsx.

## Abbreviations

GBM	Glioblastoma
BBB	Blood–brain barrier
CMAP	Connectivity Map
MD	Molecular dynamics
CNS	Central nervous system
TB	Theabrownin
Jumonji C	JmjC
IC_50_	50% inhibitory concentration
CDKs	Cyclin-dependent kinases
HPLC	High-performance liquid chromatography
DEGs	Differentially expressed genes
GO	Gene Ontology
KEGG	Kyoto Encyclopedia of Genes and Genome
TNF	Tumor necrosis factor
TUNEL	Terminal deoxynucleotidyl transferase dUTP nick-end labeling
DAPI	4′, 6′-Diamidino-2-Phenylindole
CCK-8	Cell Counting Kit-8
qPCR	Quantitative polymerase chain reaction
PI	Propidium iodide
GAPDH	Glyceraldehyde 3-phosphate dehydrogenase
RMSD	Root mean square deviation
Rg	Radius of gyration
SASA	Solvent-accessible surface area
GB	Generalized Born
ΔG	Binding free energy
RMSF	Root mean square fluctuation
bEnd.3	brain endothelial cells
C6	rat glioma cells
NaFl	Sodium fluorescein
EGFR	Epidermal growth factor receptor
VEGF	Vascular endothelial growth factor
RTKs	Tyrosine kinases
PIP3	Phosphatidylinositol-3,4,5-trisphosphate
HUVECs	Human umbilical vein endothelial cells
ATCC	American Type Culture Collection
DMEM	Dulbecco’s modified Eagle medium
FBS	Fetal bovine serum
HS	Horse serum
OD	Optical density
PFA	Paraformaldehyde
PBS	Phosphate-buffered saline
RIPA	Radioimmunoprecipitation assay
