# Supplementary figures and images for: Identification of Methylstat as a Potential Therapeutic Agent for Human Glioma Cells by Targeting Cell Cycle Arrest
Source: Pharmaceuticals (Basel). 2025 Sep 8;18(9):1344. doi: 10.3390/ph18091344 (PMC12472336; doi:10.3390/ph18091344)

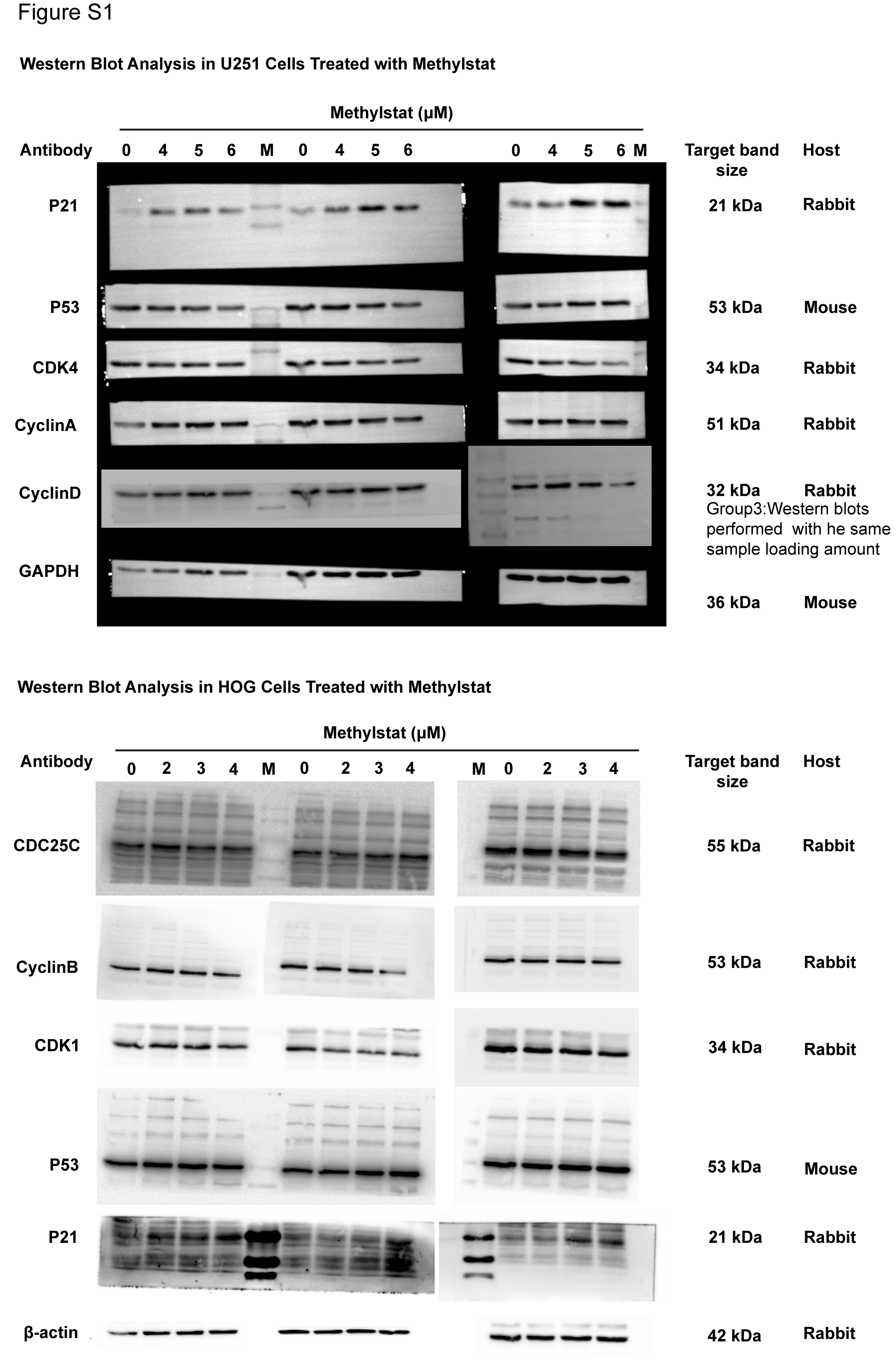

Supplement: Supplementary file 1 [file pharmaceuticals-18-01344-s001.zip › Figure S1. Western blot analysis in glioma cells treated with methylstat.tif]
